# Transanal endoscopic microsurgical submucosa dissection in the treatment of rectal adenomas and T1 rectal cancer

**DOI:** 10.1007/s00053-018-0291-3

**Published:** 2018-10-19

**Authors:** J. Baral

**Affiliations:** Klinik für Allgemein- und Viszeralchirurgie, Klinikum Karlsruhe, Moltkestr. 90, 76133 Karlsruhe, Germany

**Keywords:** Transanal surgery, Endoscopic mucosal resection, Local excision, Waterjet, Chromoendoscopy, Transanale Chirurgie, Endoskopische Mukosaresektion, Lokale Exzision, Wasserstrahl, Chromoendoskopie

## Abstract

**Background:**

The treatment of flat rectal adenomas is challenging. The technical difficulty and the potential of malignancy in suspected benign lesions are the factors in question. Surgical and interventional endoscopic techniques are implemented in Europe without a clear strategy. To minimize recurrent adenoma and unclear histopathological work up en bloc excision is desirable.

**Methods and results:**

We demonstrate in this article the transanal endoscopic microsurgical submucosa dissection (TEM-ESD) procedure as a feasible method for en bloc excision of rectal adenomas and early rectal cancer. The surgical technique is demonstrated in detail with the help of a video of the operation that is available online. The results of a consecutive series of 78 patients are presented.

**Conclusion:**

TEM-ESD is a safe procedure for resection of rectal adenomas and low risk carcinomas. It offers the possibility of organ preservation and minimizes functional disturbances. In case of a necessary salvage operation, the preserved integrity of the rectal muscle tube grants maximal oncological safety.

****Electronic supplementary material**:**

The online version of this article (10.1007/s00053-018-0291-3) includes a video on the surgical technique: TEM ESD. The article and supplemental material are available at http://www.springermedizin.de/der-chirurg. The supplemental material can be found at the end of the article under “Supplementary material”.

## Zusammenfassung

**Hintergrund:**

Flache Rektumadenome stellen aufgrund eines nicht unbeträchtlichen Entartungspotenzials und der Vielfalt der zur Verfügung stehenden Abtragungstechniken eine interdisziplinäre Herausforderung dar. Um eine klare histologische Einordnung zu ermöglichen und Rezidive zu vermeiden, muss das Ziel aller Techniken die vollständige Abtragung der Adenome sein.

**Methoden und Ergebnisse:**

Die chirurgisch-operativen und flexibel-endoskopischen Verfahren entwickelten sich in Europa unabhängig voneinander. Ihr Einsatz erfolgte bisher nicht nach klaren strategischen Vorgaben. In diesem Beitrag wird die Operationsmethode der transanal-endoskopischen mikrochirurgischen Submukosadissektion (TEM-ESD) zur En-bloc-Abtragung von großen Rektumadenomen und kleinen Rektumkarzinomen vorgestellt. Durch die Kombination der TEM-Operationstechnik mit den interventionellen endoskopischen Techniken (Chromoendoskopie, Wasserstrahl) steht damit ein neues und technisch einfaches Verfahren zur Verfügung. Anhand eines Operationsvideos, welches online zur Verfügung steht, wird die Operationstechnik detailliert dargestellt. Ergänzend werden die Ergebnisse einer Fallserie von 78 Patienten vorgestellt.

**Schlussfolgerung:**

Die TEM-ESD stellt ein sicheres Verfahren zur Entfernung von Rektumadenomen und Low-risk-Rektumkarzinomen dar, ermöglicht den Organ- und Funktionserhalt des Rektums und bietet die größtmögliche onkologische Sicherheit für den Fall einer notwendigen Salvage-Operation.

Since the development of modern surgery, operations on the rectum have presented a challenge for the surgeon. The narrow pelvis with its bony surroundings and resulting poor visibility during surgery meant that even small tumors located favorably (as we would say today) in the central rectum in many cases resulted in the loss of the rectum. This was just as true for benign lesions as it was for malignant tumors.

For this reason, scientists developed flexible endoscopic and transanal techniques to allow for the minimally invasive treatment of lesions. The spectrum of flexible endoscopy techniques ranges from endoscopic polypectomy using the cautery snare to EMR (endoscopic mucosa resection), pEMR (piecemeal endoscopic mucosa resection) and ESD (endoscopic submucosa dissection), all of which are wall-preserving techniques, as well as full-thickness resection using the FTRD (full-thickness resection device) system [15].

Of the transanal techniques, the transanal endoscopic microsurgery (TEM) procedure developed by Professor Buess in the 1980s has established itself and is today used with a number of different surgical platforms from various manufacturers [4]. This method is most often used for full-thickness resection, but since it can be done bimanually, the technique also allows for a differentiated dissection in the rectal wall layers.

The small rectal carcinoma with low risk of lymph node metastasis (G1/2, T1, L0, V0) can be successfully treated through local resection while preserving the organ. When looking at the indications to determine which technique to use against rectal cancer, a major problem is posed by the limited number of pretherapeutic options available for detecting lymphovascular invasion and deep submucosal infiltration [6].

The same applies to the question as to whether lesions that were thought to be benign before surgery do in fact contain occult carcinomas. If such lesions are not resected en bloc, the quality of the histopathological evaluation will be limited and, in the case of an exposed carcinoma, it will then not be possible to reliably assess the risks with respect to local control and to metastatic spread. In certain cases, this necessitates completion surgery with rectum resection and TME (total mesorectal excision), something that would not be required in the case of an en bloc resection with good risk estimation. Because of this, en bloc resection of all suspect masses in the colorectum is desirable.

As a full-thickness resection method, TEM is in cases of benign and malignant rectal lesions a safe technique with high R0 resection and success rates. However, it may also be associated with an increased rate of sphincter loss in cases requiring completion surgery, primarily where lesions in the lower rectum are involved [2, 7].

Looking at the flexible techniques for en bloc excision we note that ESD in the colorectum is a technically challenging, lengthy and hence cost-intensive procedure with a long learning curve [12]. As such, this technique is also used selectively in the colorectum.

A new technique yet to be established should solve the following problems associated with the procedures used to date: the risk to the sphincter in case completion surgery is required following full-thickness resection using the TEM technique; with ESD, the technical difficulties and the long learning curve, especially in cases of large adenomas and local complications (recurrence, scarring following previous removal attempts, etc.).

The combination of ESD with classic TEM to create TEM ESD is an attempt to overcome the aforementioned disadvantages of the various techniques and to enable regular treatment of such lesions in the rectum.

## Diagnostics

Small carcinomas and adenomas in the colorectum are in most cases diagnosed during prophylactic check-ups and then treated. In the pre-operative phase, standard diagnostic procedure includes a complete colonoscopy to determine the condition of the residual colon. The decision as to which technique to use to remove the tumor should be based on a best possible assessment of the dignity, location and extent of the lesion. Lesions in the lower rectum that can be reached during the digital rectal examination are additionally classified according to Mason [9].

Assessing the dignity of extensive flat rectal adenomas is difficult. With sufficient clinical expertise, it is useful to know the endoscopic aspect of the lesion, including an evaluation of its morphology (growth type, loss of surface structure, tissue vulnerability, tissue hardness, vascularization, etc.). Japanese studies that applied this evaluation revealed a high level of accuracy in detecting deep infiltrating (i. e., no longer curative), locally resectable submucosa carcinomas (sensitivity 94.9%, specificity 76% [16]).

In cases of flat adenomas, a mucosa classification (pit pattern) and the classification of the surface structure and growth type (Paris classification) should be done to estimate the cancer risk. Chromoendoscopic (indigo carmine) and virtual techniques are also applied [17].

Further diagnostic procedures in cases of suspected cancer include transrectal endosonography and thin-layer MRI. Biopsies should not be performed on patients with flat adenomas prior to a planned endoscopic removal since scarring could impair the interventional removal.

## Instrument set and equipment

**Fig. 1:**
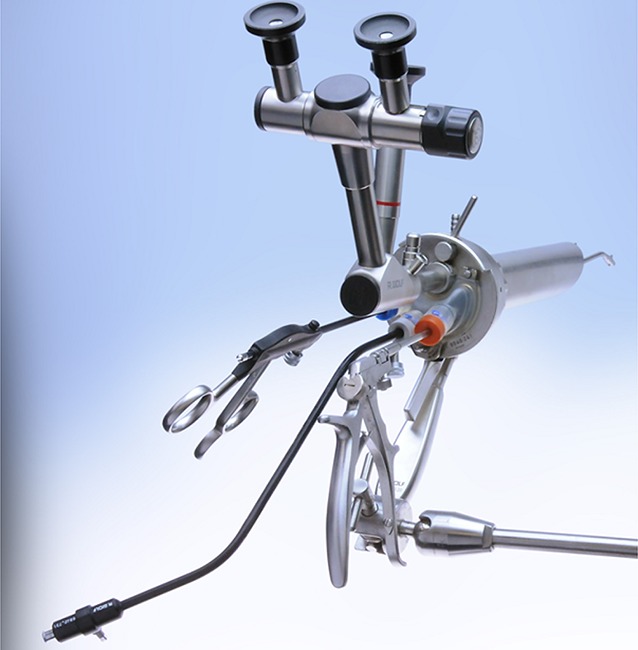
Surgical equipment for transanal endoscopic microsurgery (TEM). (Reproduced with permission from Richard Wolf GmbH, Knittlingen, Germany. This content is not part of the Open Access license.)

The TEM instrument set developed by Buess consists of a surgical rectoscope that can accept tubes of different lengths to modularly adapt to the available working height in the rectum. The surgical rectoscope is affixed to the operating table by means of a supporting arm (Fig. 1).

The rectoscope seals off the rectum in the anal canal to create a closed space with a constant and pressure-controlled pneumorectum. The TEM pump unit creates and controls the pneumorectum. Arising vapors and secretions are evacuated by means of continuous suction. HD optics give the surgeon an excellent view of the surgical area. The irrigation channel integrated into the optical system allows the user to clean the lenses and flush the surgical area.

The TEM instruments include scissors, diathermic needles and self-righting needle holder as well as variously configured grasping forceps. All of the instruments used in the field of minimally invasive surgery can also be employed for TEM interventions. A needle knife with monopolar current is used for excision. For full-thickness excision, sealing instruments (ultrasound scissors) are used to reliably control bleeding.

**Fig. 2:**
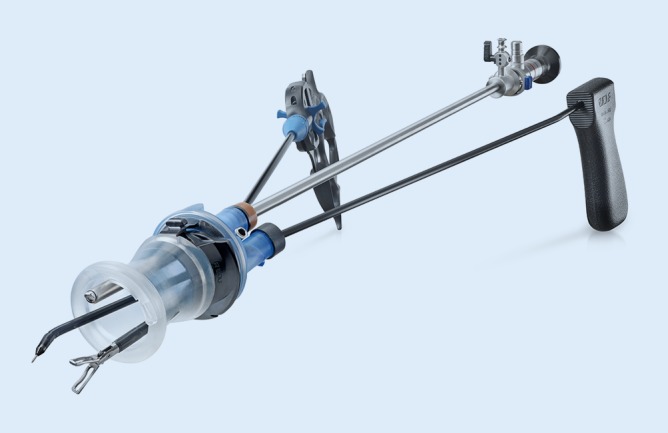
The KeyPort flex. (Reproduced with permission from Richard Wolf GmbH, Knittlingen, Germany. This content is not part of the Open Access license.)

Further surgical platforms for transanal surgery are available from various manufacturers. The primary difference lies in the system used to fix the operating tube onto the operating table and in the way the optical system is guided. When using fixed platforms, the surgeon can perform the surgery without further assistance. When using set-ups that do not have the supporting arm fixed to the operating table, an assistant is required to control the movements of the tube and the optics. These systems are modifications of surgical platforms used in single-port surgery. The advantage here is that the camera can be guided flexibly and independently of the surgical rectoscope (Fig. 2).

## Surgical technique

**Fig. 3:**
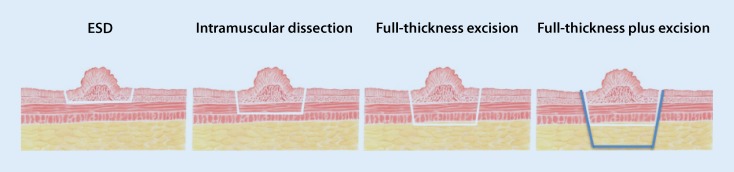
Dissection planes in the rectal wall. *ESD* endoscopic submucosa dissection. (From [10]. This content is not part of the Open Access license.)

With its bimanual preparation technique, transanal endoscopic microsurgery (TEM) permits dissection in the anatomically determined layers of the rectal wall (mucosectomy, submucosal dissection, intramuscular dissection, full-thickness excision, full-thickness plus excision; Fig. 3).

**Fig. 4:**
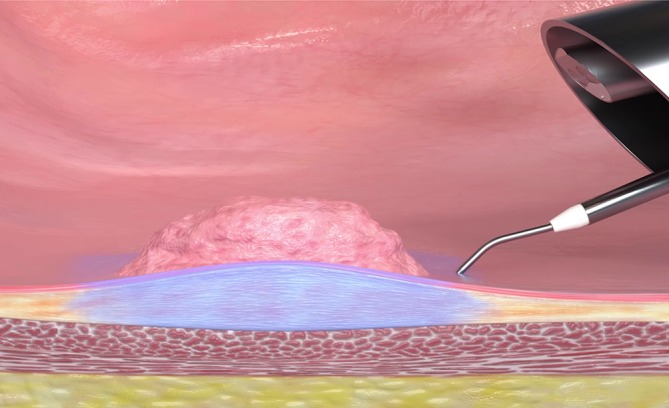
Waterjet applicator with chromoendoscopy. (Reproduced with permission from Erbe Elektromedizin GmbH, Tübingen, Germany. This content is not part of the Open Access license.)

With TEM ESD, submucosal dissection with chromoendoscopy is done by applying the technique described by Yamamoto and using the TEM instrument set [18]. TEM ESD was performed with a waterjet and chromoendoscopy for the first time in 2008 ([3]; Fig. 4).

### Important aspects of presurgical consultation

Consultation is done using the standard information sheets for transrectal surgery. An increased level of incontinence is not expected following the surgery. It is important to point out to the patient that late afterbleeding could occur from the residual wound surface in the rectum.

### Preparing for surgery

For transanal endoscopic interventions, positioning the patient for best access to the rectal lesion to be removed greatly facilitates the surgery.

**Fig. 5:**
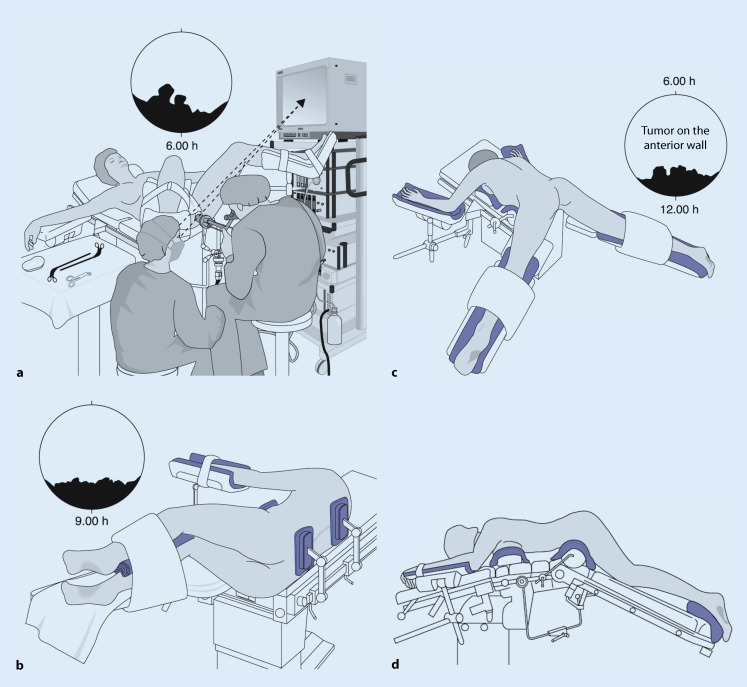
Positioning. **a** Lithotomy position, **b** lateral position, **c**, **d** Westhues position. (Reproduced with permission from Richard Wolf GmbH, Knittlingen, Germany. This content is not part of the Open Access license.)

The principal extension of the lesion to be removed should be positioned downward during the surgery. The patient should be placed accordingly into the lithotomy position, lateral position or Westhues position (Fig. 5). Intestinal cleansing is done by means of orthograde bowel irrigation as is done in preparation for colonoscopies. The intervention is performed under administration of single-shot antibiotics.

### Surgical technique

Once the patient is positioned under full anesthesia and complete muscle relaxation has occurred, the surgeon cautiously performs digital sphincter dilatation. Then the surgical rectoscope in the length selected for the surgery is guided into the anal canal and the rectum is inspected using the inserted glass window and manual air insufflation. During this phase, the patient can be repositioned as required to better expose the lesion.

The surgical rectoscope is now fastened to the operating table with the support arm, the glass window is replaced with the optics attachment and the system connected to the insufflator and camera. After creating the pneumorectum through CO_2_ insufflation and inserting the instruments, the surgical platform is adjusted as desired and the actual surgery can begin.

For full-thickness excision, the rectum wall is severed as vertically as possible in a circular fashion around the lesion and into the perirectal fat tissue. In order to maintain an overview of the situation in the narrow rectum area, visible blood vessels must be prophylactically coagulated and any bleeding that occurs stopped immediately. To help the surgeon here, all of the instruments used in minimally invasive surgery are available (monopolar and bipolar coagulation, ultrasound scissors, clip techniques and sutures).

Preparation is done from distal to proximal. The surgical specimen is then recovered through the opening provided in the surgical rectoscope. The resulting wall defect is carefully rinsed and closed with a transverse full-thickness suture. Both ends of the suture are secured with a titanium clip.

**Fig. 6:**
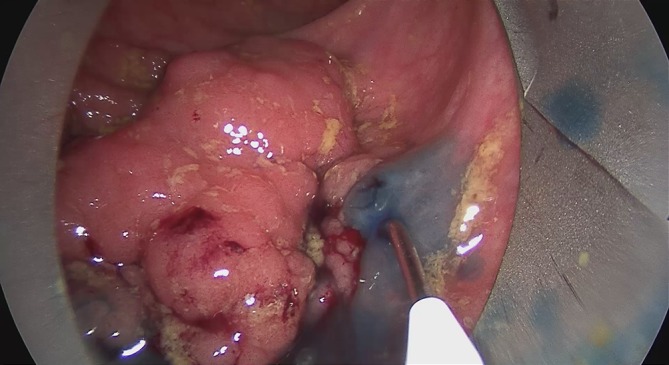
Transanal endoscopic microsurgery endoscopic submucosa dissection (TEM ESD): Use of the waterjet applicator (Waterjet, Erbe Elektromedizin GmbH) to form a submucosal edema in the tela submucosa. Good delimitation of adenomatous tissue achieved with indigo carmine for intravital staining (chromoendoscopy). (From [10]. This content is not part of the Open Access license.)

The surgical steps for TEM ESD differ from those of full-thickness excision only in the preparation depth (Fig. 6).

Using the waterjet and waterjet applicator in combination with chromoendoscopy with indigo carmine allows for a precise discrimination of the extent of the lesion (Fig. 6). The formation of a submucosal edema with distinct staining of the submucosa results in excellent tissue layer contrasting.

Preparation begins at the distal end of the mass to be removed. Severing the mucosa and submucosa provides access to the preparation layer on the muscularis propria. Then, under repeated waterjet application to provide intravital staining and to create the submucosal edema, preparation is done tight along the muscle layer up to the cranial end of the lesion while observing the free circumferential resection margin.

**Fig. 7:**
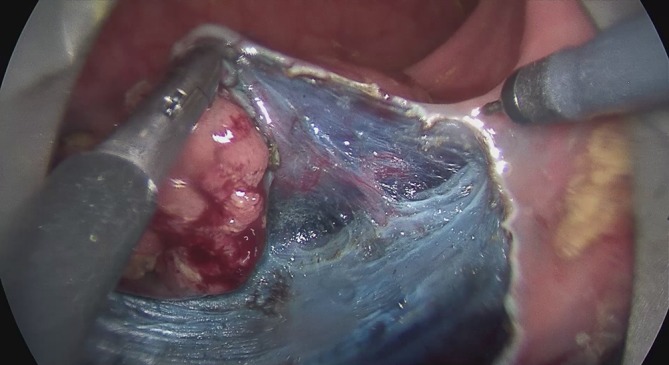
Transanal endoscopic microsurgery endoscopic submucosa dissection (TEM ESD): monopolar dissection with a needle knife (VIO® 3 Generator, Erbe Elektromedizin GmbH). Differentiated color contrast of the tissue layers and submucosal vessels by intravital staining with indigo carmine (chromoendoscopy). (From [10]. This content is not part of the Open Access license.)

**Fig. 8:**
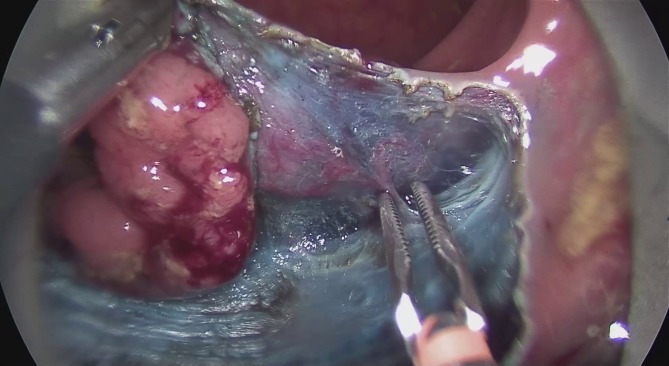
Transanal endoscopic microsurgery endoscopic submucosa dissection (TEM ESD): submucosal vessels are prophylactically cauterized and severed with bipolar coagulation to control bleeding (VIO® 3 Generator, Erbe Elektromedizin GmbH). (©Dr. J. Baral, Clinic for General and Visceral Surgery, Klinikum Karlsruhe. This content is not part of the Open Access license.)

**Fig. 9:**
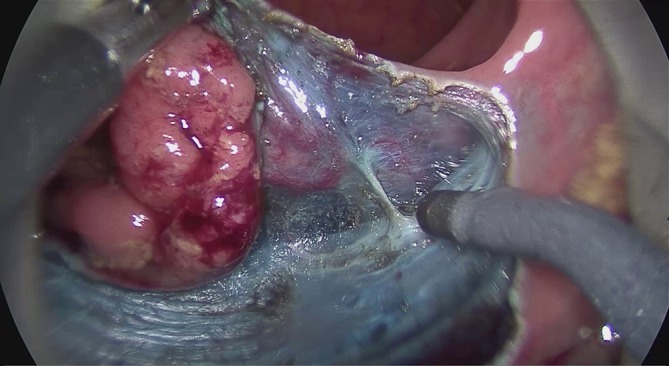
Transanal endoscopic microsurgery endoscopic submucosa dissection (TEM ESD): monopolar dissection of the muscular lamina with complete resection of the tela submucosa. (©Dr. J. Baral, Clinic for General and Visceral Surgery, Klinikum Karlsruhe. This content is not part of the Open Access license.)

The intravital staining with indigo carmine allows the surgeon on the one hand to discern the boundary between dysplastic and normal tissue and on the other to find the precise preparation depth on the muscular layer. The vessels found in the tela submucosa are highlighted by the color contrast provided by the intravital staining and prophylactically cauterized and severed with bipolar coagulation (Figs. 7, 8 and 9).

The diathermic needle is used to cut very close to the muscularis propria. Muscle fibers removed along with the resected tissue serve to confirm the complete resection of the submucosal layer. This becomes especially important if a rectal carcinoma is revealed during the examination of the specimen. Complete resection of the submucosa allows for an exact determination of the infiltration depth of the tumor and a precise estimation of the risk of metastasis. If a deeper dissection is required due to adhesions, this is done in a controlled manner using the diathermic needle. If wall defects or an accidental opening of the abdominal cavity occur, these can be immediately air sealed with an intrarectal suture using the instrument set.

**Fig. 10:**
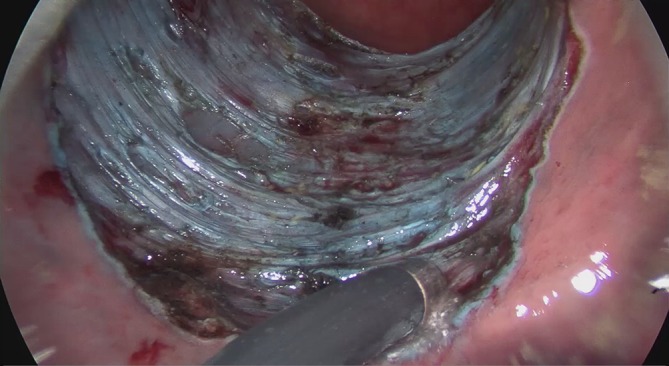
Transanal endoscopic microsurgery endoscopic submucosa dissection (TEM ESD): final check of the excision plane and prophylactic monopolar coagulation of the stumps of the vessels and the resection margins to control bleeding and avoid postoperative bleeding. (From [10]. This content is not part of the Open Access license.)

The excision and removal of the lesion is followed by the inspection of the resection margins and the careful check of the excision plane. Monopolar and bipolar coagulation is performed to reduce the risk of postoperative bleeding (Fig. 10).

**Fig. 11:**
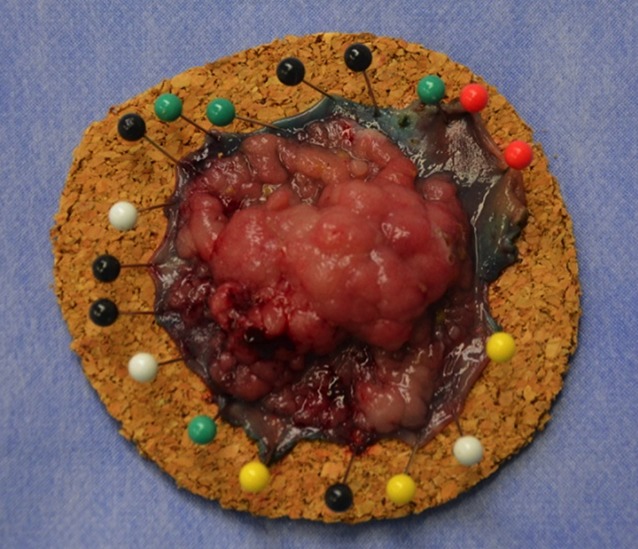
Affixation of the specimen for histopathological analysis. (From [10]. This content is not part of the Open Access license.)

In patients in whom the rectal muscle tube is intact, defect closure is not required following transanal endoscopic submucosal dissection. The specimen is affixed to cork and sent for histopathological analysis (Fig. 11). The en bloc excision of the lesion enables the exact microscopic assessment of both the circumferential and basal resection margins.

**Fig. 12:**
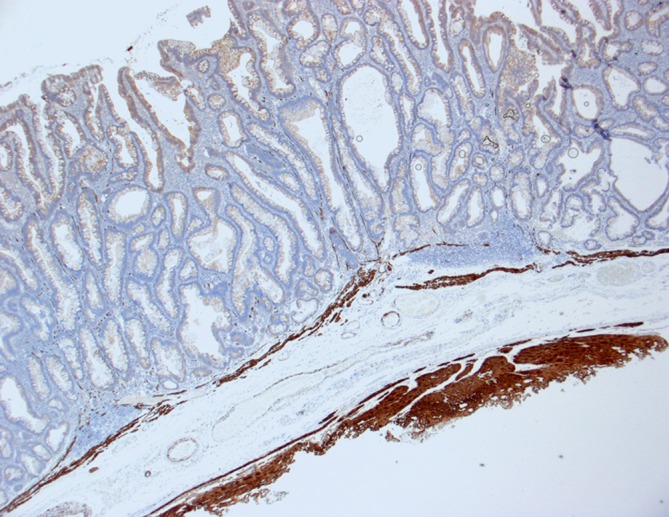
Histochemical staining for desmin: clear visualization of the completely resected tela submucosa by staining of the resected muscle fibers of the muscularis propria. (From [10]. This content is not part of the Open Access license.)

Where the tela submucosa is excised completely (visibly indicated by the basal fibers of the muscularis propria removed along with the tissue), it is possible to precisely determine the depth of tumor infiltration in the submucosal layer and to stratify the risk of metastasis to regional lymph nodes (Fig. 12). The histological examination is performed based on the guidelines of the Japanese Society of Gastroenterology [11].

### Postoperative care

Depending on the anesthesia used, oral diet can begin postoperatively on the day of surgery. Patients report slight peranal blood discharge during the first days following surgery. A temporary, moderate increase in body temperature is often observed. If the inflammation parameters are normal and there is no sign of fever, the patient can, depending on the extent of the surgery, be released to outpatient care starting on postoperative day 2. If signs of inflammation persist or if high fever occurs, antibiotic therapy is applied.

When the first endoscopic examination is performed depends on the extent of the resection and the clinical symptoms. If following circumferential ESD, for example, a high risk of stenosis exists in the surgical area, the initial examination should take place within 6 weeks at the latest.

## Aftercare

Aftercare is provided according to the type of lesion removed. For fully resected (R0) adenomas, aftercare is done in accordance with the S3 guideline for polypectomy [6]. In cases of histopathologically uncertain R0-resected adenomas, endoscopic checks are performed after 3, 6 and 12 months.

For locally excised low-risk carcinomas there are no clear national recommendations concerning the sequence, duration and type of aftercare. Following case-by-case risk assessment, we look for orientation to the recommendations of the colorectal carcinoma working group in Great Britain ([10]; Table 1).

**Table 1:** Aftercare in case of carcinoma

Years post-op	1^a^	2	3	4	5
Rectoscopy/Endosonography^a^	3/6/12	6/12	6/12	6/12	6/12
Colonoscopy	X	–	–	X	–
CT Thorax	6/12	1	1	1	1
MRI	3/12	6/12	1	1	1
CEA	3/12	6/12	1	1	1

*CEA* Carcinoembryonic antigen

^a^Figures indicate months following surgery

One particular difficulty in these cases involves the monitoring of the mesorectal compartment, the lymph nodes localized here and any existing tumor deposits or venous invasion by tumor cells. For this reason, MRI scans must be performed regularly in addition to the rectoscopic and endosonographic examinations.

## Results

In the period from Jan. 1, 2015, to Dec. 31, 2017, we performed the TEM ESD surgery on 78 patients. The acquired data are being prospectively collected and analyzed in a database.

The criteria for indication were as follows:

1. All lesions in the rectum had a diameter > 2 cm and/or could not be cleanly resected en bloc by endoscopic means,
2. Deep infiltration (deeper than the submucosa) ruled out by macroscopic and endosonographic or MRI morphological diagnosis.

Location of the excised lesions are listed in Table 2, while the histological findings are available in Table 3.

**Table 2:** Location of excised lesions in the rectum

	*N* (%)
Upper rectum (>11 cm from anus)	9 (12)
Middle rectum (6–10 cm from anus)	41 (54)
Lower rectum (0–5 cm from anus)	26 (34)
Anterior wall	19 (25)
Posterior wall	30 (39.5)
Right	13 (17)
Left	14 (18.5)

The duration of the surgery averaged 49.2 min (range: 4–231 min). No relevant loss of blood was seen during the surgery; no transfusions were required.

**Table 3:** Histopathological findings

	*N*
**Lesion type**	
*Adenoma*	66 (87%)
With high grade IEN	25
With low grade IEN	41
*Carcinoma*	10 (13%)
pT1 sm1	3
pT1 sm2	3
pT1 sm3	2
pT2	2
**Size of lesion**	
*Max. diameter (average in mm)*	39.2
Range (mm)	9–170
*Min. diameter (average in mm)*	31.5
Range (mm)	7–145

*IEN* intraepithelial neoplasia

**Table 4:** Postoperative complications

*No complications*	70 (92%)
*Bleeding*	4 (5%)
With endoscopic hemostasis	3
With surgical hemostasis	1
*Perforation/abscess*	0
*Other infection*	1 (1.5%)
*Rectal stenosis*	1 (1.5%)
*Dindo-Clavien I*	1
*Dindo-Clavien II*	1
*Dindo-Clavien IIIa*	3
*Dindo-Clavien IIIb*	1
*Dindo-Clavien IV*	0
*Dindo-Clavien V*	0

*Dindo-Clavien* classification according to Dino-Clavien

In all cases it was possible to perform a clean resection macroscopically. The basal resection margin was free of tumors in all adenomas. In 15 cases, however, the lateral resection margin could not be evaluated due to small lacerations, making it impossible to histopathologically verify clean resection (Table 3).

Eight carcinomas were histopathologically entirely removed. In 2 cases the basal part of the resection margin was afflicted by a tumor. The first case involved a pT1 sm1 tumor, so that a follow-up resection in the form of a TEM full-thickness resection was sufficient. In the second case we had a T2 tumor, indicating a deep anterior rectal resection with total mesorectal excision.

In 70 cases (92%) no complications were experienced in the postoperative period. Postoperative bleeding occurred in 4 cases. Of these, 3 cases required endoscopic hemostasis while one required operative revision. Blood transfusion was not required in any case. No perforations or abscesses occurred. One patient developed pneumonia postoperatively. A non-relevant rectal stenosis was detected endoscopically in one patient. The complications that occurred are summarized in Table 4 along with their Dindo-Clavien classification.

The entire inpatient stay lasted on average 3.1 days (range: 1–8).

## Discussion

The latest recommendations for the treatment of rectal adenomas and small rectal carcinomas were presented in the current guidelines of the DGVS (Deutsche Gesellschaft für Gastroenterologie) and in the Colorectal Carcinoma Guideline [5, 6]. The aim of polyp removal is a polyp-free intestine with reduced carcinoma risk and a minimum of complications. This means that all polyps with the potential of degeneration must be completely removed.

The removal of flat adenomas poses particular difficulties. The difficulty in achieving local R0 removal and the risk of occult cancer in the adenoma both increase in correlation with the size and morphology of the adenoma. For polyps > 5 mm snare excision should be performed with or without injection and using diathermy current. For flat adenomas, removal should be carried out using the EMR (endoscopic mucosa resection) technique. This can be performed successfully en bloc on adenomas up to 2 cm in size and with a low recurrence rate if R0 resection is achieved. For larger adenomas it is recommended to perform piecemeal EMR.

Since there is a strong correlation between R0 resection and the recurrence rate, piecemeal EMR is associated with a 30% higher risk of recurrence [1, 8].

Besides the difficulties of histopathological examination in cases where a rectal carcinoma is incidentally found in an adenoma removed using the piecemeal technique, these patients will also need to be operated on again to have the recurrent adenoma removed.

In the end, the risk of adenoma recurrence remains over the long term. The patients must be strictly monitored [13]. It is thought that these incomplete polypectomies are responsible for 20% of interval carcinomas [14].

According to the DGVS guidelines, ESD is not an established resection technique for colorectal lesions due to the high complexity of the intervention, the highly variable success rate and the increased risk of complications, and should therefore be left for specialized centers to perform as part of scientific studies. [5]. In Japan, various techniques for assessing the surface of target lesions have been developed (Kickuchi, Paris, Sano, etc.) and a differential treatment algorithm defined for deciding when a lesion can be removed en bloc (ESD) or with pEMR, or whether it should be primarily handled using a surgical method with lymphadenectomy [17].

As a transanal full-thickness technique, TEM has in the rectum a higher success and R0 resection rate than does ESD. In the analysis by Arezzo, an en bloc and R0 resection rate of 98.7% was seen for a total of 1407 patients from 9 case series treated with the TEM full-thickness technique. This compares to 87.8% seen in the ESD group (9 case series, 490 patients). For the patient material analyzed, this meant that abdominal completion surgery was required less often following TEM. The authors concluded from this that TEM represents the gold standard in Europe for treating extensive rectal adenomas [2]. However, where completion surgery is required following local excision in the lower rectum, an increased rate of sphincter loss as well as reduced TME quality must be expected due to the full-thickness defect that occurred [7].

In our own patients with rectal adenomas, TEM ESD has a success rate of nearly 100% and a macroscopic R0 resection rate of 100%. Due to lateral lacerations, this could only be histologically confirmed in 77% of cases. The basal resection margin, however, was tumor-free in all cases. There were no recurrences following adenoma resection and low-risk carcinoma excision (median follow-up 12 months). The complication rate was moderate (bleeding requiring intervention 5%, perforation 0%). In the resected tissue, 13% of carcinomas were incidental findings. The low-risk carcinomas were, despite the applied ESD technique, removed R0 in 80% of cases and it was possible to get a good histopathological estimation of their metastasis risk.

Combining the TEM surgical technique with interventional endoscopic techniques (ESD, chromoendoscopy, waterjet) provides us with a new procedure that, at least in the rectum, unites the advantages of the two procedures (TEM, ESD) and minimizes their disadvantages. Flat adenomas can be removed with no major difficulties en bloc and without full-thickness defects. Scarring left from previous treatments does not present any major problems. In adenoma cases the complication rate is low and the recurrence rate nearly zero. For submucosal carcinomas, the high en bloc resection rate allows for precise histopathological risk assessment for locoregional metastasis. In cases of required rectum resection, no increased rate of sphincter loss is to be expected.

## Practical conclusion

- TEM ESD opens up the possibility of the minimally invasive and en bloc removal of flat rectal adenomas regardless of their extension and regardless of any previous treatments.
- In cases of adhesions following previous endoscopic interventions, deeper dissection can be conducted in a controlled manner.
- If perforations occur in the muscle tube, these can be closed in a controlled manner by suturing.
- In the distal rectum, small rectal carcinomas can be resected as an excision biopsy while safely preserving the compartment.
- The muscle tube of the rectum wall is preserved with TEM ESD.
- If a follow-up surgery is required, there is no reason to expect a higher rate of sphincter loss and the TME quality is not impaired.

## Caption Electronic Supplementary Material

ESM: Video on the TEM ESD surgical technique—English version
